# Draft genome sequence of *Staphylococcus epidermidis* strain 33-2 isolated from the human nasal cavity

**DOI:** 10.1128/mra.00208-26

**Published:** 2026-04-23

**Authors:** Ken-ichi Okuda

**Affiliations:** 1 Department of Life, Environment and Applied Chemistry, Fukuoka Institute of Technology 53335, Fukuoka, Japan; Fluxus Inc., Sunnyvale, California, USA

**Keywords:** *Staphylococcus epidermidis*, draft genome, coagulase-negative staphylococci, biofilm, opportunistic infection

## Abstract

Here, I report the draft genome sequence of *Staphylococcus epidermidis* strain 33-2 isolated from the human nasal cavity. The genome assembly consists of three contigs with a total length of 2,576,776 bp and an average GC content of 32.0%. Genome annotation identified 2,382 protein-coding sequences, 19 rRNA genes, and 60 tRNA genes.

## ANNOUNCEMENT

*Staphylococcus epidermidis* is a commensal of the human skin and nasal microbiota but is also a frequent cause of device-associated and opportunistic infections (1–6). To provide genomic information for a colonizing isolate, we sequenced *S. epidermidis* strain 33-2 isolated from a self-collected nasal swab from a healthy adult volunteer who provided informed consent.

The specimen was collected in Tokyo, Japan, in October 2017 and was cultured immediately after collection. For primary isolation, the swab tip was directly streaked onto Brain Heart Infusion (BHI) agar and incubated aerobically at 37°C for 24 h. A single colony was restreaked once on fresh BHI agar to obtain a pure culture and then inoculated into BHI broth and grown at 37°C overnight. An aliquot of the broth culture was supplemented with glycerol (final concentration, 15%) and stored at −80°C.

Genomic DNA was extracted from cells harvested from 5 mL of an overnight BHI broth culture at 37°C using the Wizard Genomic DNA Purification Kit (Promega) according to the manufacturer’s instructions. DNA purity (A260/280 and A260/230, both 1.8) was assessed using a NanoDrop spectrophotometer, DNA concentration was determined using the Quant-iT dsDNA BR Assay Kit (Invitrogen), and DNA fragmentation was evaluated by agarose gel electrophoresis (100 ng dsDNA loaded) prior to library construction; these results indicated that the DNA met the quality criteria for library preparation.

Genomic DNA (6.7 µg) was sheared to ~20 kb using a Covaris g-TUBE, and a ~20 kb SMRTbell library was prepared using the SMRTbell Template Prep Kit 1.0 (Pacific Biosciences). DNA fragments were size-selected using the BluePippin system (Sage Science) with a 15-kb cutoff to enrich fragments >15 kb. Sequencing was performed on the PacBio RS II platform using the DNA/Polymerase Binding Kit P6 v2 and DNA Sequencing Reagent 4.0 on SMRT Cell 8Pac v3 with MagBead Kit v2 and MagBead OneCellPerWell (Pacific Biosciences). The subread N50 was 14,537 bp.

Sequencing generated 99,188 polymerase reads and 129,089 subreads, yielding 1,393,143,778 bp of sequence data and providing 540× genome coverage. Reads were processed in SMRT Analysis v2.3.0 to generate filtered subreads (minimum polymerase read quality, 0.80; minimum polymerase read length, 100 bp; and minimum subread length, 500 bp) prior to assembly. *De novo* genome assembly was performed using the HGAP Assembly v.3 pipeline implemented in SMRT Analysis v2.3.0 (7). The assembly was further processed using Circlator v1.2.1 (8), and consensus polishing was performed with Quiver (RS_Resequencing.1) in SMRT Analysis v2.3.0 by remapping subreads to curated contigs (7). Default parameters were used except where otherwise noted.

The final draft genome assembly consists of three contigs totaling 2,576,776 bp, with an overall GC content of 32.0%. The assembly N50 was 2,443,506 bp, and no assembly gaps were detected. Genome annotation using DFAST (9) identified 2,382 protein-coding sequences, 19 rRNA genes, and 60 tRNA genes. No CRISPR arrays were detected, and the coding density was 82.9% (Table 1). Genome completeness was not estimated. The genome sequence reported here provides a useful resource for future comparative genomic and functional studies of nasal *S. epidermidis* isolates.

**TABLE 1:** Genome features of *S. epidermidis* strain 33-2

Feature	Value
Total length (bp)	2,576,776
Number of contigs	3
GC content (%)	32.0
Assembly N50 (bp)	2,443,506
Protein-coding sequences	2,382
rRNA genes	19
tRNA genes	60
CRISPR arrays	0
Coding ratio (%)	82.9

## Data Availability

The draft genome sequence of *Staphylococcus epidermidis* strain 33-2 has been deposited in DDBJ/ENA/GenBank under the accession numbers BAAJXV010000001–BAAJXV010000003. The BioProject and BioSample accessions are PRJDB40243 and SAMD01815340, respectively. The PacBio sequencing reads have been submitted to the DDBJ Sequence Read Archive under accession number DRR902767. The DDBJ Sequence Read Archive is a public repository providing integrated access to raw sequencing data within the International Nucleotide Sequence Database Collaboration (INSDC) (10).
